#  Changes in Bone Mineral Density After Prophylactic Bilateral Salpingo-Oophorectomy in Carriers of a *BRCA* Mutation

**DOI:** 10.1001/jamanetworkopen.2019.8420

**Published:** 2019-08-07

**Authors:** Joanne Kotsopoulos, Elizabeth Hall, Amy Finch, Hanxian Hu, Joan Murphy, Barry Rosen, Steven A. Narod, Angela M. Cheung

**Affiliations:** 1 Women’s College Research Institute, Toronto, Ontario, Canada; 2 Dalla Lana School of Public Health, University of Toronto, Toronto, Ontario, Canada; 3 Osteoporosis Program, University Health Network, Centre of Excellence in Skeletal Health Assessment, University of Toronto, Toronto, Ontario, Canada; 4 Department of Gynecology Oncology, Princess Margaret Cancer Center, Toronto, Ontario, Canada

## Abstract

**Importance:**

Preventive surgery is strongly recommended for individuals with a *BRCA* mutation at a young age to prevent ovarian cancer and improve overall survival. The overall effect of early surgical menopause on various health outcomes, including bone health, has not been clearly elucidated.

**Objective:**

To evaluate the association of prophylactic bilateral salpingo-oophorectomy with bone mineral density (BMD) loss among individuals with a *BRCA* mutation.

**Design, Setting, and Participants:**

This retrospective cohort study of participants with a *BRCA* mutation who underwent oophorectomy through the University Health Network, Toronto, Ontario, Canada, recruited participants from January 2000 to May 2013. Eligibility criteria included having a *BRCA* mutation, at least 1 ovary intact prior to surgery, and no history of any cancer other than breast cancer. Bone mineral density was measured using dual-energy x-ray absorptiometry before and after surgery. Data analysis began in December 2018 and finished in January 2019.

**Main Outcomes and Measures:**

The annual change in BMD from baseline to follow-up was calculated for the following 3 anatomical locations: (1) lumbar spine, (2) femoral neck, and (3) total hip.

**Results:**

A total of 95 women had both a baseline and postsurgery BMD measurement with a mean (SD) follow-up period of 22.0 (12.7) months. The mean (SD) age at oophorectomy was 48.0 (7.4) years. Among women who were premenopausal at time of surgery (50 [53%]), there was a decrease in BMD from baseline to follow-up across the lumbar spine (annual change, −3.45%; 95% CI, −4.61% to −2.29%), femoral neck (annual change, −2.85%; 95% CI, −3.79% to −1.91%), and total hip (annual change, −2.24%; 95% CI, −3.11% to −1.38%). Self-reported hormone therapy use was associated with significantly less bone loss at the lumbar spine (−2.00% vs −4.69%; *P* = .02) and total hip (−1.38% vs −3.21; *P* = .04) compared with no hormone therapy use. Among postmenopausal women at time of surgery (45 [47%]), there was also a significant decrease in BMD across the lumbar spine (annual change, −0.82%; 95% CI, −1.42% to −0.23%) and femoral neck (annual change, −0.68%; 95% CI, −1.33% to −0.04%) but not total hip (annual change, −0.18%; 95% CI, −0.82% to 0.46%).

**Conclusions and Relevance:**

This study found that oophorectomy was associated with postoperative bone loss, especially among women who were premenopausal at the time of surgery. Targeted management strategies should include routine BMD assessment and hormone therapy use to improve management of bone health in this population.

## Key Points

**Question:**

What is the association of preventive oophorectomy with bone health in individuals with a *BRCA* mutation?

**Findings:**

In this cohort study of 95 women with a *BRCA* mutation, prophylactic oophorectomy was associated with a decline in bone mineral density, which was most apparent among women who were premenopausal at surgery. Use of hormone therapy was associated with less bone loss.

**Meaning:**

Although limited by the small sample size, these findings support targeted management strategies to maintain bone health in this high-risk population.

## Teaser

This cohort study evaluates the association of prophylactic bilateral salpingo-oophorectomy with bone mineral density loss among women with a *BRCA* mutation.

## Introduction

Individuals with a deleterious mutation in 1 of 2 breast cancer susceptibility genes, *BRCA1* (OMIM 113705) or *BRCA2 *(OMIM 600185), face a high lifetime risk of developing ovarian cancer, estimated to be 49% for a *BRCA1 *mutation and 21% for a *BRCA2 *mutation.^1^ Risk-reducing or prophylactic bilateral salpingo-oophorectomy (ie, surgical removal of ovaries and fallopian tubes) is recommended given that effective screening or chemoprevention options are currently lacking for this high-risk population. Based on the current National Comprehensive Cancer Network guidelines, preventive surgery is recommended for individuals aged 35 to 40 years who have a *BRCA1* mutation and for individuals aged 40 to 45 years with a *BRCA2* mutation.^2^

Many individuals with a *BRCA* mutation will undergo surgical menopause prior to natural menopause and face the consequences of abrupt ovarian hormonal withdrawal.^3,4^ Established adverse effects of early surgical menopause include vasomotor symptoms, sexual functioning, heart disease, and declines in attention and memory.^5^ Of interest in this study is the association of early menopause with bone health. Endocrine-induced loss of bone mineral density (BMD) across the menopausal transition has been well established in the general population. The association of oophorectomy with BMD is most evident among those who were premenopausal at the time of surgery, with a rapid decline within the first 2 years after surgery that appears to stabilize over time.^6,7^ To our knowledge, few studies have evaluated the association of preventive surgery with BMD loss in individuals with a *BRCA1* or *BRCA2* mutation. Although limited by their cross-sectional nature, collectively the data suggest elevated rates of bone disease following oophorectomy, especially among those who were premenopausal at the time of surgery.^8,9,10,11,12,13^ There are no standardized guidelines for the management of bone health in this population after surgery. Individuals with a *BRCA* mutation may be at an elevated risk of BMD decline because of a history of cancer. Furthermore, important emerging data^14^ suggest significant dysregulation in progesterone-mediated receptor activator of nuclear factor κΒ signaling in these women.

To our knowledge, there have been no longitudinal studies evaluating postoophorectomy changes in BMD specifically among individuals with a *BRCA1* or *BRCA2* mutation. Thus, the overall goal of this study was to evaluate the association of preventive oophorectomy and abrupt hormonal withdrawal with BMD loss among individuals with a *BRCA* mutation and to investigate the extent to which this association is modified by menopausal status at surgery and exogenous hormone use.

## Methods

### Study Population

The study population included all women who elected to undergo prophylactic bilateral salpingo-oophorectomy at the University Health Network and Women’s College Hospital, Toronto, Ontario, Canada, from January 2000 to May 2013.^15^ Eligibility criteria included the following: (1) having a documented *BRCA* mutation, (2) being aged 30 to 75 years, (3) having at least 1 intact ovary prior to surgery, and (4) having no history of cancer other than breast cancer. Eligible participants were recruited by mail, followed by a telephone call the month prior to surgery. After providing written informed consent, participants were asked to complete a medical release form as well as 3 research questionnaires as follows: (1) medical history questionnaire, (2) Menopause-Specific Quality of Life Intervention questionnaire, and (3) Sexual Activity Questionnaire. Women were required to have BMD assessments at baseline (presurgery) and after surgery or according to the Ministry of Health Guidelines in Ontario. All women who completed the baseline questionnaires before surgery were recontacted by mail or telephone to complete follow-up questionnaires at approximately 1 and 3 years after completion of their baseline questionnaires. The institutional review board of the Women’s College Hospital, Toronto, approved the study. This study follows the Strengthening the Reporting of Observational Studies in Epidemiology (STROBE) reporting guideline.

### Data Collection

The medical history questionnaire was designed specifically for this study and asked participants to report on their reproductive history, surgical history, height, weight, menopausal status, cancer history, and medication use, including hormone therapy (HT). Participants also reported on lifestyle factors, including smoking status and physical activity. Women were also asked to provide detailed information on their personal history of breast cancer as well as their use of aromatase inhibitors and selective estrogen receptor modulators (SERMs).

### Dual-Energy X-ray Absorptiometry Assessment

Women were required to have a baseline dual-energy x-ray absorptiometry (DXA) assessment prior to surgery and were advised to undergo follow-up assessments approximately 1 year after surgery or according to the Ministry of Health Guidelines in Ontario. Bone mineral density measurements were completed by DXA at the University Health Network, Women’s College Hospital, or at referring centers in Ontario, which used their own DXA scan equipment and institutional imaging protocols. A BMD measurement within 2 months before surgery was categorized as a baseline measurement, and a BMD measurement 6 or more months after surgery was categorized as a follow-up BMD measurement. For women with multiple postsurgery DXA scans, comparisons were made using the first follow-up DXA scan available. All BMD measurements were converted to Hologic-equivalent values using standard reference formulas.^16,17^ Bone mineral density was reported as grams per centimeter squared and by T score across the following 3 anatomical locations: (1) lumbar spine, (2) femoral neck, and (3) total hip. Based on the World Health Organization guidelines for diagnosis of osteoporosis in postmenopausal women, we classified women with a T score less than −2.5 as having osteoporosis and participants with a T score between −2.5 and −1 as having osteopenia.^18^ The Canadian-specific Fracture Risk Assessment Tool^19^ was used to calculate 10-year risk of hip fracture according to each patient’s femoral neck BMD, age, body mass index (calculated as weight in kilograms divided by height in meters squared), smoking history, and alcohol intake.

### Patient Selection

The study recruitment is outlined in eFigure 1 in the Supplement. A total of 320 women undergoing prophylactic salpingo-oophorectomy at the University Health Network were invited to participate in a study evaluating the association of surgical menopause with various outcomes, as outlined by Hall et al.^20^ Of the 320 women who were approached, 201 (62.8%) consented to participate. Of the 201, 79 (39.3%) were excluded for various reasons, including 4 (5.1%) who declined to participate during the follow-up period, 16 (20.2%) who had a secondary or recurrent malignant neoplasm, 1 (1.3%) who tested negative for a *BRCA* mutation, and 20 (25.3%) who were lost to follow-up. Among the remaining 160 women, 54 (33.8%) were excluded because they did not have a baseline (n = 38 [70.4%]) or follow-up (n = 16 [29.6%]) DXA scan. A total of 95 participants were eligible for inclusion in the final analysis. For the current analysis, only women who had a baseline and at least 1 follow-up DXA scan conducted at the same center, on the same machine, and using the same measurement procedure were eligible for inclusion.

### Statistical Analysis

The McNemar test for correlated proportions was used to evaluate changes in medical and lifestyle characteristics of study participants between baseline and follow-up. A paired *t *test was used to compare body mass index at time of baseline and follow-up DXA. The annual change in BMD was expressed as the percentage change in BMD (100 × [follow-up BMD − baseline BMD] / baseline BMD) divided by the time between the baseline and follow-up BMD measurements in years.

Owing to our small sample size, we stratified participants a priori into binary categories for each exposure of interest. Unpaired *t* tests were used to evaluate differences in BMD T scores between subgroups (eg, menopausal status at time of surgery, HT use following surgery, and prior breast cancer). Use of SERMs (eg, tamoxifen or raloxifene), aromatase inhibitors (eg, exemestane or anastrozole), and bisphosphonate (eg, risedronate or alendronate) were reported in each participant’s medical health questionnaire. Current use of HT, SERMs, or other medications was defined as use in the months from surgery to follow-up DXA. For subgroup analyses, regular supplement users were defined as women taking both calcium and vitamin D supplements. Physical activity status was determined according to each participant’s response to 2 questions about the intensity and duration of physical activity performed per week. Women who reported moderate or strenuous physical activity for at least 3 to 5 hours per week were designated as highly physically active. All *P* values were based on 2-sided tests and considered statistically significant if *P* ≤ .05. Statistical analyses were performed using SAS version 9.1.3 (SAS Institute).

## Results

The baseline characteristics of the 95 women with a *BRCA1* or *BRCA2* mutation who were included in this analysis are summarized in Table 1. There were 50 women (53%) who were premenopausal prior to surgery, and 45 women (47%) who were postmenopausal prior to surgery. Overall, the mean (SD) age at surgery was 48.0 (7.4) years, 44.0 (4.2) years among premenopausal women and 52.4 (7.7) years among postmenopausal women. There were 43 women (45%) with a history of breast cancer (14 premenopausal women [28%] and 29 postmenopausal women [64%]), and 32 (34%) were previously treated with chemotherapy. A mean (SD) of 22.0 (12.7) months elapsed between surgery and the first follow-up DXA scan.

**Table 1.** Demographic Characteristics and Clinical Features of Study Participants Stratified by Menopausal Status at Time of Surgery

Characteristic	Participants With DXA at Baseline and Follow-up, No. (%)		
	All Participants (N = 95)	Premenopausal Women (n = 50)	Postmenopausal Women (n = 45)
Mutation status			
* BRCA1*	47 (49)	30 (60)	17 (38)
* BRCA2*	48 (51)	20 (40)	28 (62)
Age at time of surgery, mean (SD), y	48.0 (7.4)	44.0 (4.2)	52.4 (7.7)
<40	10 (11)	9 (18)	1 (2)
40-44	27 (28)	21 (42)	6 (13)
45-49	24 (25)	14 (28)	10 (22)
50-54	16 (17)	6 (12)	10 (22)
55-60	13 (14)	0	13 (29)
>60	5 (5)	0	5 (11)
Follow-up period, mean (SD), mo^a^	22.0 (12.7)	21.3 (12.0)	22.9 (13.6)
Procedure			
Bilateral salpingo-oophorectomy only	16 (17)	5 (10)	11 (24)
Bilateral salpingo-oophorectomy and hysterectomy	79 (83)	45 (90)	34 (76)
Parity			
0	16 (17)	10 (20)	6 (13)
1-2	53 (56)	28 (56)	25 (56)
≥3	26 (27)	12 (24)	14 (31)
Menopause			
Natural	17 (18)	0	17 (38)
Prior hysterectomy	10 (11)	0	10 (22)
Medication^b^	18 (19)	0	18 (40)
Prior breast cancer	43 (45)	14 (28)	29 (64)

Abbreviation: DXA, dual-energy x-ray absorptiometry.

^a^ Refers to time from surgery to follow-up DXA scan.

^b^ Includes menopause induced by chemotherapy.

Table 2 is a summary of the change in various medical and lifestyle factors among the study participants before and after surgery. There was an increase in the proportion of women who used HT following oophorectomy (3 [3%] vs 27 [28%]; *P* < .001). This was largely owing to the increase in HT use by women who were premenopausal before surgery (0 vs 23 [46%]; *P* < .001). Among the 23 who initiated HT, 4 (17%) had a history of breast cancer. There was also a significant increase in the proportion of women who used a SERM following surgery (15 [16%] vs 21 [22%]; *P* = .004), and this was for the most part limited to women who were postmenopausal at the time oophorectomy (13 [29%] vs 15 [52%]; *P* = .008). There was a significant increase in the proportion of women who used calcium (38 [45%] vs 67 [77%]; *P* < .001) and vitamin D supplements (41 [45%] vs 68 [78%]; *P* < .001) after surgery. This increase was similar among premenopausal and postmenopausal women. There was a significant increase in weight after surgery; however, this was limited to women who were postmenopausal at the time of surgery. Based on the World Health Organization classification of bone disease, 39 women (41%) in our cohort were classified as having osteopenia and 4 (4%) as having osteoporosis before surgery (Table 2). These proportions increased significantly to 51 women (54%) with osteopenia (*P* < .001) and 6 (6%) with osteoporosis (*P* < .001) after surgery. The increase in participants with osteopenia was most apparent among women who were premenopausal at the time of surgery (17 [34%] vs 26 [52%]; *P* = .002). Fracture Risk Assessment Tool scores were low (<6%) for 10-year risk of hip fracture, regardless of menopausal status at time of surgery (data not shown).

**Table 2.** Changes in Medical and Lifestyle Characteristics of Women Between Baseline and Follow-up

Characteristic	No. (%)								
	All Participants (N = 95)			Premenopausal Women (n = 50)			Postmenopausal Women (n = 45)		
	Baseline	Follow-up	*P* Value^a^	Baseline	Follow-up	*P* Value	Baseline	Follow-up	*P* Value
HT use									
Current^b^	3 (3)	27 (28)	<.001	0	23 (46)	<.001	3 (7)	4 (9)	NA
Previous	2 (2)	1 (1)	NA	0	0 (0)	NA	2 (4)	1 (2)	NA
SERM use^c^									
Current^b^	15 (16)	21 (22)	.004	2 (4)	6 (12)	NA	13 (29)	15 (33)	.008
Previous	9 (9)	12 (13)	NA	6 (12)	3 (6)	NA	3 (7)	9 (20)	NA
AI use^d^									
Current^b^	6 (6)	9 (9)	NA	2 (4)	3 (6)	NA	4 (9)	6 (13)	NA
Previous	0	0	NA	0	0	NA	0	0	NA
Bisphosphonate use^e^									
Current^b^	3 (3)	10 (11)	NA	0	3 (6)	NA	3 (7)	7 (16)	NA
Previous	2 (2)	1 (1)	NA	0	0	NA	2 (4)	1 (2)	NA
Smoking status, cigarettes/d									
0	84 (88)	85 (89)	NA	44 (88)	43 (86)	NA	40 (89)	42 (93)	NA
1-5	8 (8)	9 (9)	NA	4 (8)	6 (12)	NA	4 (9)	3 (7)	NA
Not reported	3 (3)	1 (1)	NA	2 (4)	1 (2)	NA	1 (2)	0	NA
Alcohol intake, drinks/wk									
0-3	71 (75)	73 (77)	NA	36 (72)	38 (76)	NA	35 (78)	35 (78)	NA
4-9	15 (16)	14 (15)	NA	9 (18)	7 (14)	NA	6 (13)	7 (16)	NA
>9	6 (6)	6 (6)	NA	3 (6)	4 (8)	NA	3 (7)	2 (4)	NA
Not reported	3 (3)	2 (2)	NA	2 (4)	1 (2)	NA	1 (2)	1 (2)	NA
Intensity of physical activity									
Little	18 (19)	20 (21)	NA	7 (14)	10 (20)	NA	11 (24)	10 (22)	NA
Moderate	59 (62)	61 (64)	NA	33 (66)	30 (60)	NA	26 (58)	31 (69)	NA
Strenuous	10 (11)	10 (11)	NA	7 (14)	8 (16)	NA	3 (7)	2 (4)	NA
Not reported	8 (8)	4 (4)	NA	3 (6)	2 (4)	NA	5 (11)	2 (4)	NA
Duration of physical activity, h/wk									
<1	9 (9)	11 (12)	NA	3 (6)	5 (10)	NA	6 (13)	6 (13)	NA
1-3	36 (38)	30 (32)	NA	15 (30)	12 (24)	NA	21 (47)	18 (40)	NA
3-5	20 (21)	30 (32)	NA	14 (28)	21 (42)	NA	6 (13)	9 (20)	NA
>5	22 (23)	20 (21)	NA	15 (30)	10 (20)	NA	7 (16)	10 (22)	NA
Not reported	8 (8)	4 (4)	NA	3 (6)	2 (4)	NA	5 (11)	2 (4)	NA
Supplement use^f^									
Calcium	38 (40)	67 (71)	<.001	17 (42)	35 (76)	<.001	21 (47)	32 (71)	.007
Vitamin D	41 (43)	68 (72)	<.001	21 (44)	37 (80)	<.001	20 (44)	31 (69)	.004
Weight, mean (SD), kg	66.94 (11.34)	67.75 (11.59)	.04	65.49 (11.53)	66.18 (11.37)	.28	68.56 (11.03)	69.51 (11.70)	.04
DXA result									
Normal	52 (55)	38 (40)	<.001	31 (62)	21 (42)	.002	21 (47)	17 (38)	.13
Osteopenia	39 (41)	51 (54)		17 (34)	26 (52)		22 (49)	25 (56)	
Osteoporosis	4 (4)	6 (6)		2 (4)	3 (6)		2 (4)	3 (7)	

Abbreviations: AI, aromatase inhibitors; DXA, dual-energy x-ray absorptiometry; HT, hormone therapy; NA, not applicable; SERM, selective estrogen receptor modulators.

^a^ *P* values compare baseline with follow-up values and were derived using McNemar test for correlated proportions. *P* values comparing body mass index were derived using the *t* test for paired samples.

^b^ Defined as use in the months between surgery and follow-up.

^c^ Includes use of tamoxifen or raloxifene as reported in the medical health questionnaire.

^d^ Includes use of anastrozole, letrozole, or exemestane as reported in the medical health questionnaire.

^e^ Includes use of risedronate, alendronate, and etidronate.

^f^ Defined as intake of supplements in the past year as reported in the medical health questionnaire.

**Figure 1.**
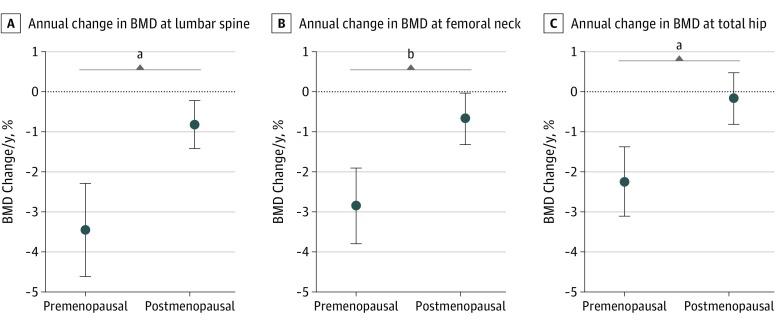
Annual Change in Bone Mineral Density (BMD) by Menopausal Status Circles represent mean annual change, comparing BMD in grams per centimeter squared at baseline with follow-up. Whiskers represent 95% CIs. Only women with serial BMD measurements are included in the subgroup analysis. Sample sizes for each comparison are outlined in Table 3. *P* values compare mean percentage change in BMD by menopausal status and were derived using the *t* test for 2 independent samples. ^a^*P* < .001. ^b^*P* = .006.

Table 3 summarizes the baseline and follow-up T scores by menopausal status at surgery, while Figure 1 summarizes the annual change in BMD in the same women. Among women who were premenopausal at surgery, there was a decrease in the BMD across the lumbar spine (annual change, −3.45%; 95% CI, −4.61% to −2.29%), femoral neck (annual change, −2.85%; 95% CI, −3.79% to −1.91%), and total hip (annual change, −2.24%; 95% CI, −3.11%, to −1.38%) after oophorectomy (Figure 1). Among women who were postmenopausal at surgery, there was a decrease in BMD across the lumbar spine (annual change, −0.82%; 95% CI, −1.42% to −0.23%) and femoral neck (annual change, −0.68%; 95% CI, −1.33% to −0.04%) but not for the total hip (annual change, −0.18%; 95% CI, −0.82% to 0.46%) after oophorectomy (Figure 1).

**Table 3.** Comparison of BMD T Scores at Baseline and Follow-up by Menopausal Status at Surgery and HT Use After Surgery

Time	Mean BMD T Score (95% CI)					
	Menopausal Status			HT Use at Follow-up Among Premenopausal Women^a^		
	Premenopausal Women	Postmenopausal Women	*P* Value^b^	No HT Use	HT Use	*P* Value^b^
**Lumbar Spine**						
No.^c^	50	45	NA	27	23	NA
Baseline	−0.2 (−0.5 to 0.2)	−0.7 (−1.0 to −0.3)	.04	0.1 (−0.4 to 0.5)	−0.4 (−0.9 to 0.1)	.18
Follow-up	−0.7 (−1.0 to −0.3)	−0.8 (−1.2 to −0.4)	.65	−0.7 (−1.2 to −0.3)	−0.6 (−1.2 to 0.0)	.83
**Femoral Neck**						
No.^c^	45	42	NA	24	21	NA
Baseline	−0.4 (−0.7 to −0.1)	−0.7 (−1.0 to −0.3)	.29	−0.5 (−0.9 to −0.1)	−0.3 (−0.8 to 0.2)	.53
Follow-up	−0.7 (−1.0 to −0.4)	−0.8 (−1.2 to −0.4)	.69	−0.9 (−1.2 to −0.5)	−0.5 (−1.0 to 0.0)	.21
**Total Hip**						
No.^c^	42	29	NA	19	23	NA
Baseline	0.0 (−0.3 to 0.3)	−0.5 (−0.9 to −0.1)	.05	0.2 (−0.2 to 0.5)	−0.1 (−0.6 to 0.3)	.30
Follow-up	−0.3 (−0.6 to 0.0)	−0.6 (−1.0 to −0.3)	.11	−0.3 (−0.6 to 0.1)	−0.3 (−0.7 to 0.2)	.98

Abbreviations: BMD, bone mineral density; HT, hormone therapy; NA, not applicable.

^a^ Defined as use of HT in the months from surgery to follow-up.

^b^ *P* values compare T scores by menopausal status and HT use and were derived using the *t* test for 2 independent samples.

^c^ Only women with serial BMD measurements are included in the subgroup analysis. Sample sizes vary for women who had dual-energy x-ray absorptiometry measured at lumbar spine but not femoral neck or total hip.

Before surgery, baseline BMD as assessed by the T score in the lumbar spine and total hip was significantly lower among postmenopausal women compared with premenopausal women (lumbar spine: −0.7 [95% CI, −1.0 to −0.3] vs −0.2 [95% CI, −0.5 to 0.2]; *P* = .04; total hip: −0.5 [95% CI, −0.9 to −0.1] vs 0.0 [95% CI, −0.3 to 0.3]; *P* = .05). There were no other significant differences in the baseline or follow-up T scores between the 2 groups of women. Women who were premenopausal at surgery experienced a significantly greater annual change in BMD across the 3 anatomical sites compared with women who were postmenopausal at surgery (lumbar spine: *P* < .001; femoral neck: *P* = .006; total hip: *P* < .001) (Figure 1).

**Figure 2.**
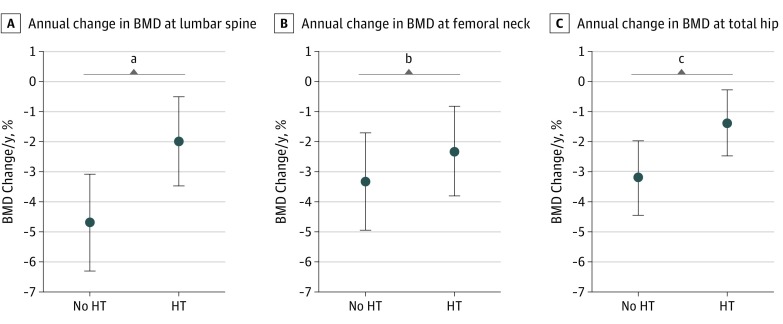
Annual Change in Bone Mineral Density (BMD) by Hormone Therapy (HT) Use After Surgery Circles represent mean annual change, comparing BMD in grams per centimeter squared at baseline with follow-up. Whiskers represent 95% CIs. Only women who were premenopausal at the time of surgery with serial BMD measurements are included in the subgroup analysis. Sample sizes for each comparison are outlined in Table 3. *P* values compare mean percentage change in BMD by HT use and were derived using the *t* test for 2 independent samples. ^a^*P* = .02. ^b^*P* = .31. ^c^*P* = .04.

We also evaluated the impact of HT after surgery among women who were premenopausal at surgery (Table 3 and Figure 2). Women who used HT had significantly less annual change in BMD than those who did not use HT at the lumbar spine (−2.00% vs −4.69%; *P* = .02) and the total hip (−1.38% vs −3.21; *P* = .04) (Figure 2). Although not statistically significant, the annual change in the femoral neck was less in women who used HT than those who did not use HT (−2.32% vs −3.32%; *P* = .31) (Figure 2). There were no significant differences in T scores by HT status (Table 3). Annual BMD changes were similar regardless of whether follow-up time was calculated from date of baseline (Figure 1 and Figure 2) or from the surgery date (eFigure 2 and eFigure 3 in the Supplement).

Findings of the analyses stratified by history of breast cancer or SERM use are summarized in eTable 1 in the Supplement, while eTable 2 in the Supplement summarizes the findings stratified by regular supplement use or physical activity. Although there appeared to be less BMD loss among women with a personal history of breast cancer and SERM use, these analyses were based on a small number of women and several comparisons so must be interpreted with caution.

## Discussion

To our knowledge, this represents the first retrospective cohort study evaluating the association of oophorectomy with bone health in *BRCA* mutation carriers using paired presurgical and postsurgical BMD measurements from the same woman. A mean (SD) of 22.0 (12.7) months after surgery, we found a decline in BMD across the lumbar spine, femoral neck, and total hip. The BMD loss was greatest among women who were premenopausal at the time of surgery and among those who did not initiate HT use after oophorectomy. Within 2 years after surgery, women who were premenopausal at the time of surgery had BMD levels similar to what was reported for women who were postmenopausal at the time of surgery; however, premenopausal women who initiated HT use had less BMD loss. Although HT minimized the amount of BMD loss, it did not completely prevent postsurgery bone loss. These findings strongly support including routine monitoring of BMD in this high-risk population and recommending adequate calcium intake, weight-bearing exercise, and the use of exogenous hormones among those without a history of breast cancer.

Given that the mean age at oophorectomy was 48 years and that 50 women (53%) were premenopausal at the time of surgery, it is not surprising that estrogen deprivation was associated with adverse effects on bone health. This has previously been reported among women in the general population undergoing oophorectomy at the time of hysterectomy.^5^ There have been few reports of postoophorectomy bone health specifically in individuals with a *BRCA1* or *BRCA2* mutation. In general, they have reported elevated rates of bone disease following surgery, especially among participants who were premenopausal before surgery.^10,11,12,13^ However, these studies were limited by their cross-sectional design, small sample sizes, and inability to compare presurgery and postsurgery BMD in the same patient.

We observed that 6 participants (6%) experienced osteoporosis, while 51 (54%) had osteopenia; the distribution was similar in women who were premenopausal and postmenopausal at surgery. These rates are consistent with those reported in previous studies of individuals at high risk of ovarian cancer owing to family history or a *BRCA* mutation.^13^ In fact, rates of bone disease or bone loss between 46% and 73% have been reported.^13^ In contrast, rates of osteopenia and osteoporosis are 49% and 7%, respectively, among individuals aged 50 to 59 years in the general population, suggesting elevated rates of osteopenia among those with a *BRCA* mutation who have undergone surgical menopause.^21^ What is striking is the consistent finding in our and prior studies^8,10,11,12^ that screening for bone disease following surgery among individuals with a *BRCA* mutation is not routine. For example, in our population, only 132 of 160 women (82.5%) had a DXA scan after surgery. Others have similarly reported suboptimal rates of DXA scans in this population.^8,10,11,12^

Loss of BMD across the menopausal transition has been well established among individuals in the general population and is attributed to a decline in endogenous ovarian hormone synthesis.^22^ For example, Greendale et al^23^ have shown a 2.5% decline in lumbar spine BMD and a 1.8% decline in femoral neck BMD during the menopausal transition period (1 year before to 2 years after final menstrual period). Interestingly, we observed higher rates of decline in our group of women who were premenopausal at surgery (−3.45% at lumbar spine and −2.85% at femoral neck). During natural menopause, ovarian senescence is gradual, beginning around age 35 years and culminating with menopause at around age 51 years. Although before menopause, the ovaries are the primary producers of bodily estrogens, following natural menopause, the ovaries continue to produce androgens (androstenedione) and testosterone, which are sources of extragonadal estrogen.^24,25^ In contrast, individuals who undergo surgical menopause, often as young as 35 years, will experience abrupt hormonal withdrawal. By exerting proapoptotic effects on osteoclasts and antiapoptotic effects on mature osteoblasts, estrogens restrain bone resorption. Thus, a lack of estrogen sends the remodeling rate into an imbalance.^22^

Currently, bilateral salpingo-oophorectomy is strongly recommended for individuals with *BRCA1* or *BRCA2* mutations to prevent ovarian cancer and has also been shown to affect overall survival.^1,26^ Interventions to reduce bone disease (and other adverse noncancer outcomes, including cardiovascular disease or cognitive decline) are necessary and may include a combination of HT use or lifestyle interventions, such as physical activity or supplement use. Importantly, the care of these individuals is further complicated by their elevated risk of developing breast cancer.^27^ Many will already have a history of breast cancer at the time of oophorectomy (as was seen in our cohort) and will not be eligible for HT to mitigate the symptoms and consequences of surgical menopause. Furthermore, treatment with endocrine disruptors, such as chemotherapy, radiation, or aromatase inhibitors, may also affect bone health.^28^

Despite the small sample size, HT use following premenopausal oophorectomy was associated with significantly less BMD decline, especially at the lumbar spine and total hip. In the current study, only 23 of 36 women (64%) who were premenopausal at the time of surgery and were candidates for HT actually initiated HT use after surgery. Given that HT is contraindicated among women with a history of breast cancer owing to fear of recurrence, it is of interest that 4 of 14 who reported HT use had a previous diagnosis of the disease. Given the important role of estrogen in maintaining various physiological functions, including bone health, this rate of HT use is far too low. In addition, we (and others) have reported improved management of menopausal symptoms (including quality of life and sexual health) with HT use following oophorectomy in the same cohort of women.^20^ Furthermore, a recent prospective study of HT use following oophorectomy^29^ found no significant association of estrogen-alone HT with breast cancer risk among individuals with a *BRCA1* mutation.

Although based on a small number of women, we observed less decline in BMD among participants with a personal history of breast cancer and with SERM use (eg, tamoxifen or raloxifene) (eTable 1 in the Supplement). This association of SERM use with slower BMD loss is consistent with existing literature^22^ that suggests that SERMs prevent osteoporosis in individuals who carry the *BRCA* mutation and undergo oophorectomy as well as reduce the risk of breast cancer.

### Limitations

This study has various limitations, including the small sample size, the use of self-reported questionnaire data (including HT use and menopausal status), and the relatively short follow-up period (22 months). This study was not sufficiently powered to further evaluate the association of other factors, such as history of breast cancer, SERM use, or physical activity, with the outcomes of interest, and we did not adjust for multiple comparisons. An additional limitation is the generalization of our findings, given that 64% of the initially eligible women received a DXA scan before and after surgery. Nevertheless, women were similar in most regards except for the use of HT following surgery (data not shown). Strengths of our study included the use of prospectively collected exposure and outcome data, a comprehensive assessment of lifestyle and medical characteristics of study participants, and availability of both presurgery and postsurgery BMD measurements conducted at 3 anatomical locations from the same woman.

## Conclusions

Results from this small analysis suggest a significant association of oophorectomy with decline in bone health that was most apparent among individuals who carry a *BRCA* mutation and underwent surgery before natural menopause. The high rates of bone loss confirm the adverse effect of instantaneous hormone loss associated with surgical menopause. Importantly, the mitigating effect of HT use (and potentially SERM use) must be considered when establishing guidelines for the management of this high-risk population with unique needs. Although longitudinal studies are necessary to evaluate the long-term effect of oophorectomy on fracture risk, our study illustrates the need to implement routine screening of bone health in this high-risk population. Of interest is the emerging importance of the receptor activator of nuclear factor κΒ–signaling pathway in *BRCA* breast cancer development and the potential chemopreventive role of anti–receptor activator of nuclear factor κΒ ligand therapy with existing agents, such as denosumab, which may have a dual role in maintaining bone health while contributing to reduction in breast cancer risk.^14^

## Notes

Supplement. **eTable 1. **Comparison of BMD and T Scores at Baseline and Follow-up by Breast Cancer History and SERM Use **eTable 2. **Comparison of BMD and T Scores at Baseline and Follow-up by Supplement Use and Physical Activity at Follow-up**** **eFigure 1. **Outline of Sample Recruitment **eFigure 2. **Annual Change in BMD (95% CI) by Menopausal Status at: (a) LS; L1-4 spine BMD; (b) FN; Femoral Neck BMD; (c) TH; Total Hip BMD sites **eFigure 3.** Annual Change in BMD (95%CI) by HT Use Following Surgery (Premenopausal Women Only) at: (a) LS; L1-4 spine BMD; (b) FN; Femoral Neck BMD; (c) TH; Total Hip BMD sites
