# Supplementary material for: Changes in Bone Mineral Density After Prophylactic Bilateral Salpingo-Oophorectomy in Carriers of a BRCA Mutation
Source: JAMA Netw Open. 2019 Aug 7;2(8):e198420. doi: 10.1001/jamanetworkopen.2019.8420 (PMC6686775; doi:10.1001/jamanetworkopen.2019.8420)
Supplement: Supplement. — eTable 1. Comparison of BMD and T Scores at Baseline and Follow-up by Breast Cancer History and SERM Use eTable 2. Comparison of BMD and T Scores at Baseline and Follow-up by Supplement Use and Physical Activity at Follow-up eFigure 1. Outline of Sample Recruitment eFigure 2. Annual Change in BMD (95% CI) by Menopausal Status at: (a) LS; L1-4 spine BMD; (b) FN; Femoral Neck BMD; (c) TH; Total Hip BMD sites eFigure 3. Annual Change in BMD (95%CI) by HT Use Following Surgery (Premenopausal Women Only) at: (a) LS; L1-4 spine BMD; (b) FN; Femoral Neck BMD; (c) TH; Total Hip BMD sites [file jamanetwopen-2-e198420-s001.pdf]

Supplementary Online Content

Kotsopolous J, Hall E, Finch A, et al. Changes in bone mineral density after prophylactic bilateral salpingo-oophorectomy in carriers of a *BRCA* mutation. *JAMA Netw Open*. 2019;2(8):e198420. doi:10.1001/jamanetworkopen.2019.8420

eTable 1. Comparison of BMD and T Scores at Baseline and Follow-up by Breast Cancer History and SERM Use

eTable 2. Comparison of BMD and T Scores at Baseline and Follow-up by Supplement Use and Physical Activity at Follow-up

eFigure 1. Outline of Sample Recruitment

eFigure 2. Annual Change in BMD (95% CI) by Menopausal Status at: (a) LS; L1-4 spine BMD; (b) FN; Femoral Neck BMD; (c) TH; Total Hip BMD sites

eFigure 3. Annual Change in BMD (95%CI) by HT Use Following Surgery (Premenopausal Women Only) at: (a) LS; L1-4 spine BMD; (b) FN; Femoral Neck BMD; (c) TH; Total Hip BMD sites

This supplementary material has been provided by the authors to give readers additional information about their work.

© 2019 Kotsopolous J et al. *JAMA Network Open.*

eTable 1. Comparison of BMD and T Scores at Baseline and Follow-up by Breast Cancer History and SERM Use [a]

	Breast Cancer History			SERM use (tamoxifen or raloxifene) [b]		
	None	Breast Cancer	*P* [c]	No SERM use	SERM use	*P*
**Lumbar Spine**	*N* = 52	*N* = 43		*N = 74*	*N = 21* [d]	
**Baseline**						
BMD in g/cm^2	1.029 (0.993, 1.065)	0.986 (0.939, 1.033)		1.016 (0.982, 1.050)	0.970 (0.910, 1.031)	
T-score	-0.2 (-0.5, 0.1)	-0.6 (-1.0, -0.2)	0.15	-0.3 (-0.6, 0.0)	-0.7 (-1.2, -0.2)	0.22
**Follow-up**						
BMD in g/cm^2	0.970 (0.931, 1.008)	0.963 (0.915, 1.011)		0.971 (0.935, 1.007)	0.950 (0.899, 1.002)	
T-score	-0.7 (-1.0, -0.4)	-0.8 (-1.2, -0.4)	0.75	-0.7 (-1.0, -0.4)	-0.9 (-1.4, -0.5)	0.37
**% Change**	-5.5 (-7.2, -3.8)*	-1.9 (-3.7, -0.1)*	**0.005**	-4.3 (-5.7, -2.8)*	-2.3 (-4.1, -0.6)*	0.08
**Femoral Neck**	*N* = 48	*N = 39*		*N = 66*	*N = 19*	
**Baseline**						
BMD in g/cm^2	0.784 (0.754, 0.813)	0.769 (0.723, 0.815)		0.786 (0.756, 0.815)	0.748 (0.698, 0.798)	
T-score	-0.5 (-0.8, -0.2)	-0.6 (-1.0, -0.2)	0.76	-0.5 (-0.7, -0.2)	-0.8 (-1.2, -0.3)	0.70
**Follow-up**						
BMD in g/cm^2	0.744 (0.715, 0.773)	0.755 (0.708, 0.802)		0.756 (0.725, 0.786)	0.724 (0.678, 0.771)	
T-score	-0.8 (-1.1, -0.5)	-0.7 (-1.1, -0.3)	0.69	-0.7 (-1.0, -0.4)	-0.9 (-1.3, -0.5)	0.69
**% Change**	-4.4 (-6.2, -2.7)*	-1.7 (-3.1, -0.3)*	**0.02**	-3.3 (-4.7, -2.0)*	-2.8 (-5.3, -0.4)*	0.11
**Total Hip**	*N = 39*	*N = 32*		*N = 57*	*N = 14*	
**Baseline**						
BMD in g/cm^2	0.970 (0.931, 1.008)	0.911 (0.869, 0.952)		0.931 (0.887, 0.974)	0.908 (0.848, 0.968)	
T-score	-0.2 (-0.5, 0.1)	-0.2 (-0.6, 0.1)	0.85	-0.2 (-0.5, 0.1)	-0.3 (-0.8, 0.2)	0.30
**Follow-up**						
BMD in g/cm^2	0.939 (0.881, 0.997)	0.895 (0.857, 0.933)		0.887 (0.857, 0.917)	0.899 (0.847, 0.952)	
T-score	-0.4 (-0.7, -0.1)	-0.4 (-0.7, 0.0)	0.99	-0.4 (-0.7, -0.2)	-0.3 (-0.8, 0.1)	0.62
**% Change**	-3.0 (-4.5, -1.5)*	-1.0 (-2.5, 0.6)	0.21	-2.9 (-4.0, -1.7)*	-0.7 (-2.8, 1.5)	0.93

© 2019 Kotsopolous J et al. *JAMA Network Open.*

[a] BMD at baseline and follow-up are presented in g/cm^2 and as T-scores. % change compares change in BMD scores in g/cm^2 between baseline and follow-up. Intervals in parenthesis describe 95% confidence intervals for each mean.
[b] SERM use is defined as current use of tamoxifen and raloxifene in the months between surgery and follow-up.
[c] *P*-values comparing BMD by breast cancer history and SERM use were derived using the Student's *t*-test for two independent samples. Significant *P*-values are bolded. *P*-values comparing BMD at baseline and follow-up were derived using the Student's *t*-test for two paired samples. Exact *P*-values for these comparisons are not shown; however, significant changes are indicated with an asterisk (*).
[d] Only women with serial BMD measurements are included in each sub-group analysis. Sample sizes vary for women who had DXA measured at lumbar spine, but not femoral neck and total hip.

© 2019 Kotsopolous J et al. *JAMA Network Open.*

eTable 2. Comparison of BMD and T Scores at Baseline and Follow-up by Supplement Use and Physical Activity at Follow-up [a]

	Regular Supplement Use [b]			Physical Activity Level [c]		
	No	Yes	P [d]	Low	High	P
**Lumbar Spine**	*N* = 31	*N* = 64		*N = 39*	*N = 48* [e]	
**Baseline**						
BMD in g/cm^2	1.013 (0.952, 1.074)	1.008 (0.976, 1.040)		0.997 (0.953, 1.041)	1.030 (0.991, 1.068)	0.33
T-score	-0.4 (-0.9, 0.1)	-0.4 (-0.7, -0.1)	0.97	-0.5 (-0.9, -0.1)	-0.2 (-0.5, 0.1)	
**Follow-up**						
BMD in g/cm^2	0.971 (0.911, 1.032)	0.964 (0.930, 0.999)		0.975 (0.928, 1.022)	0.968 (0.930, 1.006)	
T-score	-0.8 (-1.3, -0.3)	-0.7 (-1.0, -0.4)	0.89	-0.7 (-1.0, -0.3)	-0.7 (-1.0, -0.3)	0.95
**% Change**	-3.8 (-6.0, -1.7)*	-3.8 (-5.3, -2.4)*	0.78	-2.1 (-4.0, -0.3)*	-5.1 (-6.8, -3.4)*	**0.02**
**Femoral Neck**	*N* = 28	*N = 58*		*N = 38*	*N = 40*	
**Baseline**						
BMD in g/cm^2	0.763 (0.715, 0.810)	0.784 (0.754, 0.815)		0.771 (0.735, 0.807)	0.801 (0.770, 0.832)	
T-score	-0.7 (-1.1, -0.3)	-0.5 (-0.8, -0.2)	0.99	-0.6 (-1.0, -0.2)	-0.3 (-0.6, 0.0)	0.16
**Follow-up**						
BMD in g/cm^2	0.742 (0.692, 0.792)	0.752 (0.721, 0.782)		0.748 (0.713, 0.784)	0.761 (0.730, 0.792)	
T-score	-0.8 (-1.2, -0.4)	-0.7 (-1.0, -0.4)	0.86	-0.8 (-1.2, -0.4)	-0.6 (-0.9, -0.3)	0.46
**% Change**	-2.6 (-4.7, -0.5)*	-3.5 (-5.0, -2.1)*	0.69	-2.3 (-4.2, -0.4)*	-4.0 (-5.8, -2.3)*	0.19
**Total Hip**	*N = 18*	*N = 54*		*N = 29*	*N = 37*	
**Baseline**						
BMD in g/cm^2	0.884 (0.834, 0.934)	0.938 (0.893, 0.984)		0.897 (0.856, 0.937)	0.974 (0.918, 1.029)	
T-score	-0.5 (-0.9, 0.0)	-0.1 (-0.4, 0.2)	0.25	-0.4 (-0.7, 0.0)	0.1 (-0.2, 0.3)	0.09
**Follow-up**						
BMD in g/cm^2	0.877 (0.825, 0.929)	0.893 (0.863, 0.923)		0.883 (0.848, 0.919)	0.915 (0.882, 0.948)	
T-score	-0.5 (-1.0, -0.1)	-0.4 (-0.6, -0.1)	0.29	-0.5 (-0.8, -0.2)	-0.2 (-0.5, 0.0)	0.27
**% Change**	-0.8 (-3.1, 1.5)	-2.9 (-4.1, -1.8)*	0.86	-1.5 (-3.1, 0.2)	-3.2 (-4.6, -1.8)*	0.12

© 2019 Kotsopolous J et al. *JAMA Network Open.*

[a] BMD at baseline and follow-up are presented in g/cm^2 and as t-scores. % change compares change in BMD scores in g/cm^2 between baseline and follow-up. Intervals in parenthesis describe 95% confidence intervals for each mean.
[b] Regular supplement use is defined as current use of calcium and vitamin D supplements in the months between surgery and follow-up.
[c] Physical activity level is defined based on the frequency and duration of physical activity reported per week. Women at high physical activity are those who reported being at least moderately active for greater than 3hrs per week.
[d] *P*-values comparing BMD by supplement use and physical activity level were derived using the Student's *t*-test for two independent samples. Significant *P*-values are bolded. *P*-values comparing BMD at baseline and follow-up were derived using the Student's *t*-test for two paired samples. Exact *P*-values for these comparisons are not shown; however, significant changes are indicated with an asterisk (*).
[e] Only women with serial BMD measurements are included in each sub-group analysis. Sample sizes vary for women who had DXA measured at lumbar spine, but not femoral neck and total hip.

© 2019 Kotsopolous J et al. *JAMA Network Open.*

eFigure 1. Outline of Sample Recruitment.

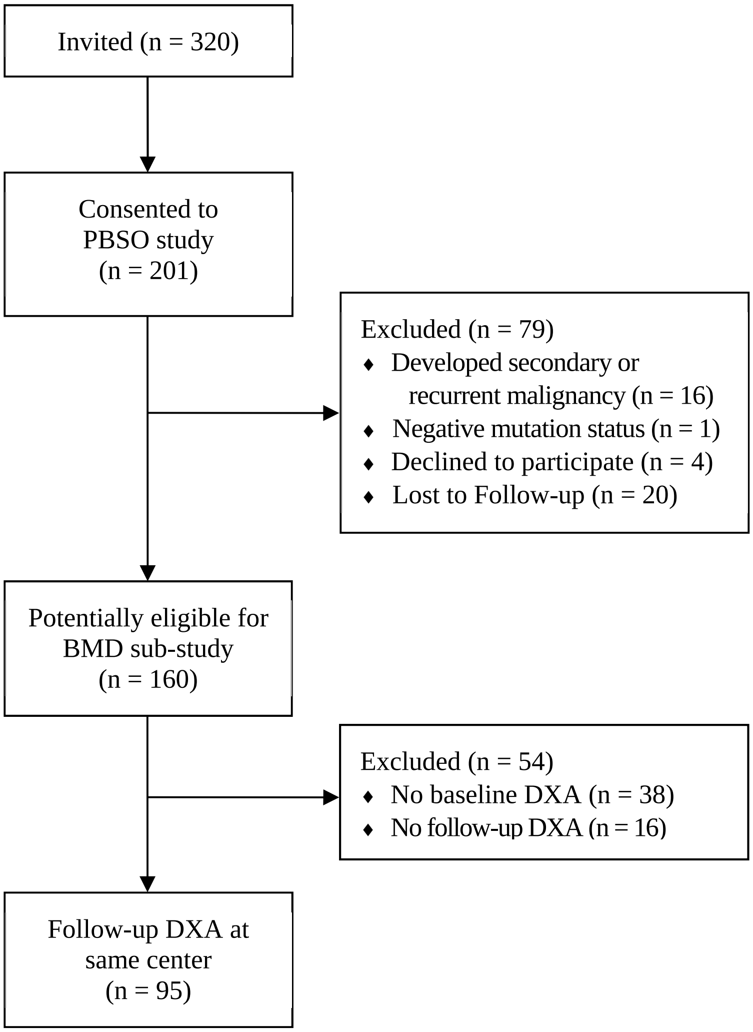

© 2019 Kotsopolous J et al. *JAMA Network Open.*

eFigure 2. Annual Change in BMD (95% CI) by Menopausal Status at: (**a**) LS; L1-4 spine BMD; (**b**) FN; Femoral Neck BMD; (**c**) TH; Total Hip BMD sites.[a, b, c]

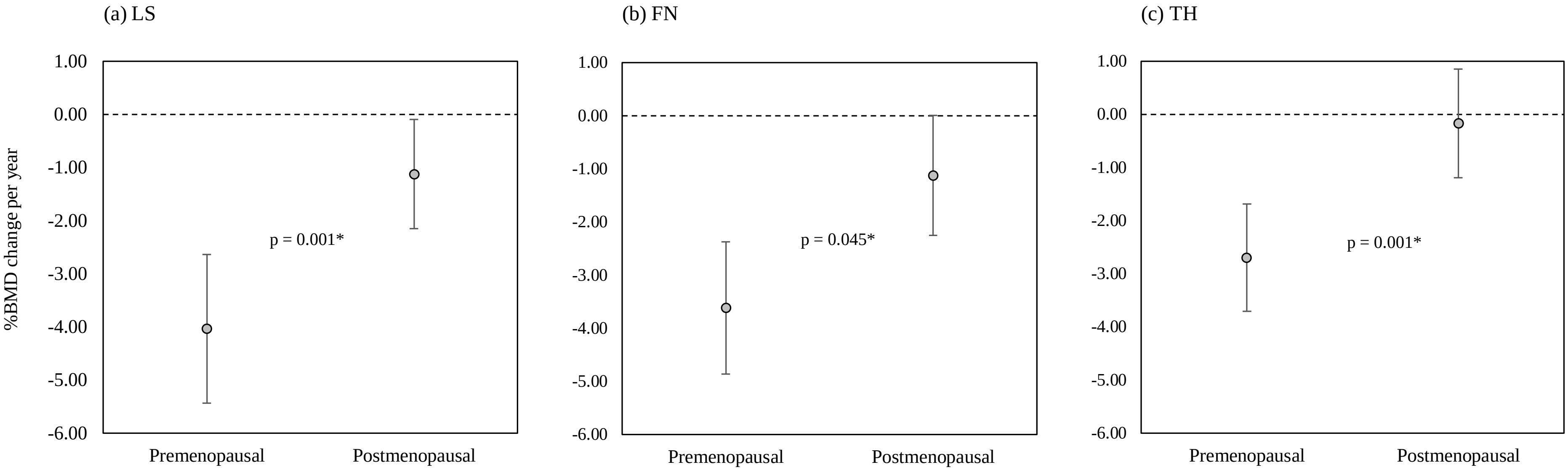

[a] Mean annual changes show % change in BMD in g/cm^2 over follow-up time since surgery. Intervals describe 95% confidence intervals for each mean.
[b] Only women with serial BMD measurements are included in each sub-group analysis. Sample sizes for each comparison are outlined in Table 3.
[c] *P*-values compare mean percent changes in BMD by menopausal status and were derived using the Student's *t*-test for two independent samples. Significant *P*-values are marked with an asterisk.

© 2019 Kotsopolous J et al. *JAMA Network Open.*

eFigure 3. Annual Change in BMD (95%CI) by HT Use Following Surgery (Premenopausal Women Only) at: (**a**) LS; L1-4 spine BMD; (**b**) FN; Femoral Neck BMD; (**c**) TH; Total Hip BMD sites.

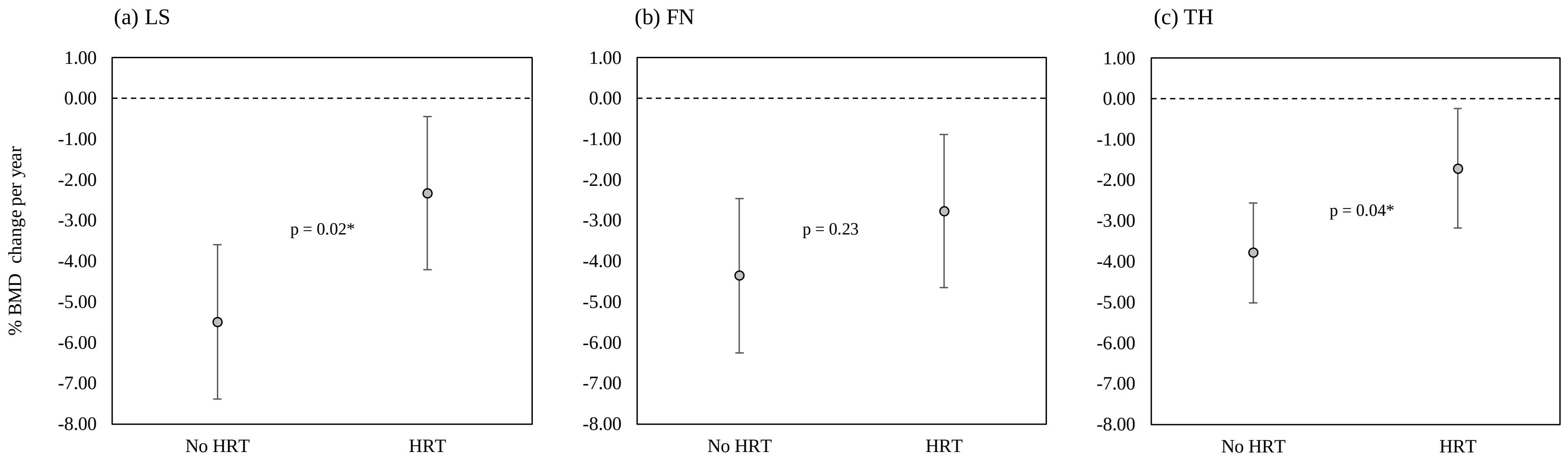

[a] Mean annual changes show % change in BMD in g/cm^2 over follow-up time since surgery. Intervals describe 95% confidence intervals for each mean.
[b] Only women with serial BMD measurements are included in each sub-group analysis. Sample sizes for each comparison are outlined in Table 3.
[c] *P*-values compare mean percent changes in BMD by HRT use and were derived using the Student's *t*-test for two independent samples. Significant *P*-values are marked with an asterisk.

© 2019 Kotsopolous J et al. *JAMA Network Open.*
